# Supplementary material for: Endothelial ADAM17 Expression in the Progression of Kidney Injury in an Obese Mouse Model of Pre-Diabetes
Source: Int J Mol Sci. 2021 Dec 25;23(1):221. doi: 10.3390/ijms23010221 (PMC8745741; doi:10.3390/ijms23010221)
Supplement: Supplementary file 1 [file ijms-23-00221-s001.zip › ijms-1485068-supplementary.pdf]

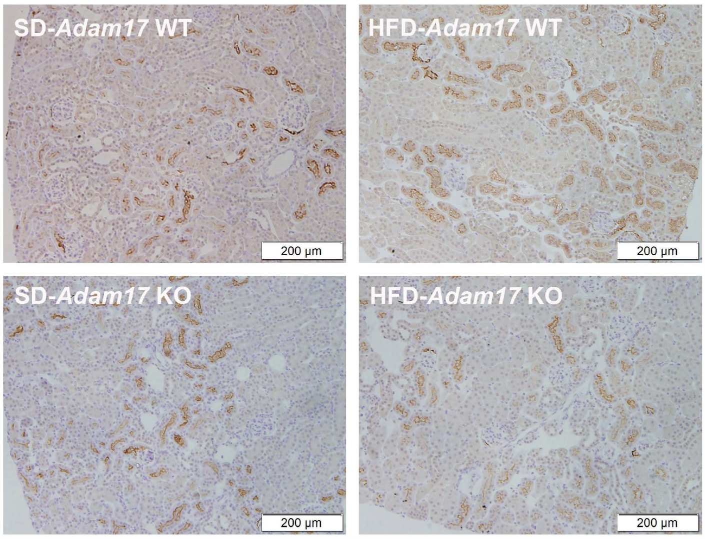


**Supplementary Figure S1:** Representative images of SGLT2 localization in the four studied groups. 100× magnification, scale bar 200 µm. Abbreviations: SD, standard diet; HFD, high fat diet; Adam17WT, wild-type; Adam17KO, knockout.

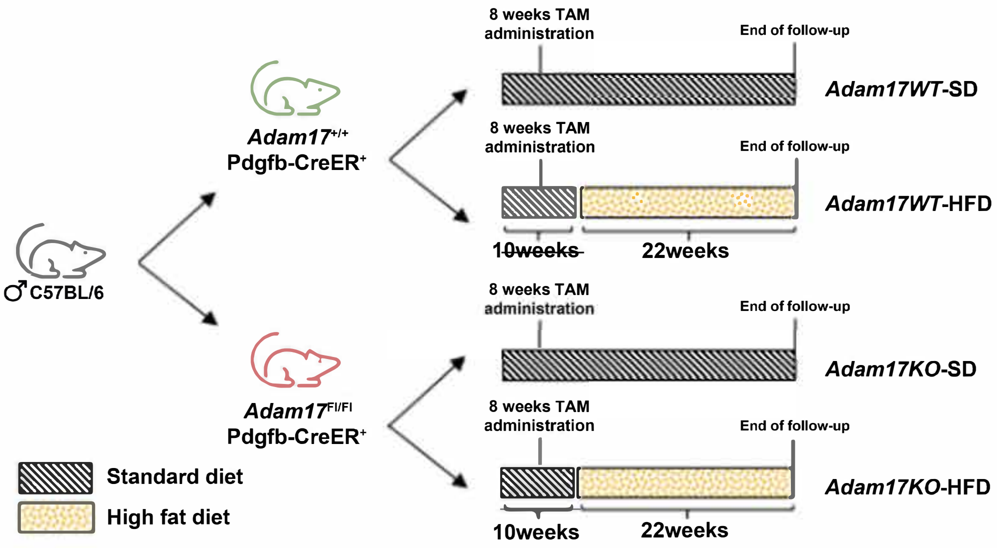


**Supplementary Figure S2:** Schematic representation of the experimental design. Male mice ca-rrying wild-type *Adam17* gene or floxed *Adam17* gene were included in the study. All animals re-ceived Tamoxifen (TAM) to induce excision of the floxed region by recombination (CreER) under the control of *Pdgf*β promoter. Abbreviations: SD, standard diet; HFD, high fat diet; Adam17WT, wild-type; Adam17KO, knockout.
